# Impact of Antioxidants on Cardiolipin Oxidation in Liposomes: Why Mitochondrial Cardiolipin Serves as an Apoptotic Signal?

**DOI:** 10.1155/2016/8679469

**Published:** 2016-05-26

**Authors:** Alexey V. Lokhmatikov, Natalia Voskoboynikova, Dmitry A. Cherepanov, Maxim V. Skulachev, Heinz-Jürgen Steinhoff, Vladimir P. Skulachev, Armen Y. Mulkidjanian

**Affiliations:** ^1^School of Physics, University of Osnabrueck, 49069 Osnabrueck, Germany; ^2^School of Bioengineering and Bioinformatics, Lomonosov Moscow State University, Moscow 119991, Russia; ^3^A.N. Belozersky Institute of Physico-Chemical Biology, Lomonosov Moscow State University, Moscow 119991, Russia; ^4^A.N. Frumkin Institute of Physical Chemistry and Electrochemistry of the Russian Academy of Sciences, Leninsky Prospect 31, Moscow 119991, Russia; ^5^Institute of Mitoengineering, Lomonosov Moscow State University, Moscow 119991, Russia

## Abstract

Molecules of mitochondrial cardiolipin (CL) get selectively oxidized upon oxidative stress, which triggers the intrinsic apoptotic pathway. In a chemical model most closely resembling the mitochondrial membrane—liposomes of pure bovine heart CL—we compared ubiquinol-10, ubiquinol-6, and alpha-tocopherol, the most widespread naturally occurring antioxidants, with man-made, quinol-based amphiphilic antioxidants. Lipid peroxidation was induced by addition of an azo initiator in the absence and presence of diverse antioxidants, respectively. The kinetics of CL oxidation was monitored via formation of conjugated dienes at 234 nm. We found that natural ubiquinols and ubiquinol-based amphiphilic antioxidants were equally efficient in protecting CL liposomes from peroxidation; the chromanol-based antioxidants, including alpha-tocopherol, were 2-3 times less efficient. Amphiphilic antioxidants, but not natural ubiquinols and alpha-tocopherol, were able, additionally, to protect the CL bilayer from oxidation by acting from the water phase. We suggest that the previously reported therapeutic efficiency of mitochondrially targeted amphiphilic antioxidants is owing to their ability to protect those CL molecules that are inaccessible to natural hydrophobic antioxidants, being trapped within respiratory supercomplexes. The high susceptibility of such occluded CL molecules to oxidation may have prompted their recruitment as apoptotic signaling molecules by nature.

## 1. Introduction

During last decades, the pathophysiological aspects of redox homeostasis came into focus of cell research; it has been shown that many diseases lead to oxidative imbalance and an increase in the level of reactive oxygen species (ROS) [1–6]. The ROS comprise the superoxide anion radical (O_2_^∙−^), hydroperoxide (H_2_O_2_), and the extremely reactive hydroxyl radical (^*∙*^OH), as well as nitrogen-containing compounds: nitroxide (NO) and peroxynitrite (ONOO^−^) [7–9]. Interaction of ROS with organic molecules could yield organic radicals, such as alkoxyl (RO^*∙*^) and peroxyl (ROO^*∙*^) radicals, which can be involved in further oxidation processes [10].

The oxidative damage to nucleic acids, proteins, and lipids, as caused by ROS, is usually considered to be a major factor in the general functional decline of tissues upon aging and age-associated degenerative diseases [2]. In case of* Metazoa*, the means to limit the ROS-induced damage seem to invoke apoptosis—the programmed cell death via activation of executor caspases and digestion of the cell from the inside [11]. Apparently, when the ROS level in a particular cell reaches a certain threshold, apoptosis is activated to eliminate the ROS-producing cell, which prevents damaging of the whole tissue; see [5, 12–14] for reviews. Apoptosis, generally useful in protecting an organism from oxidative damage, could, however, become counterproductive under conditions of an acute—although potentially reversible—oxidative stress that accompanies such pathological states as stroke and trauma. In such cases, apoptosis could lead to undesirable cell loss, cumulative tissue degradation, and ultimately even to the death of the whole organism. Therefore, a controlled prevention of apoptotic reactions under conditions of acute oxidative stress might save lives.

**Figure 1:**
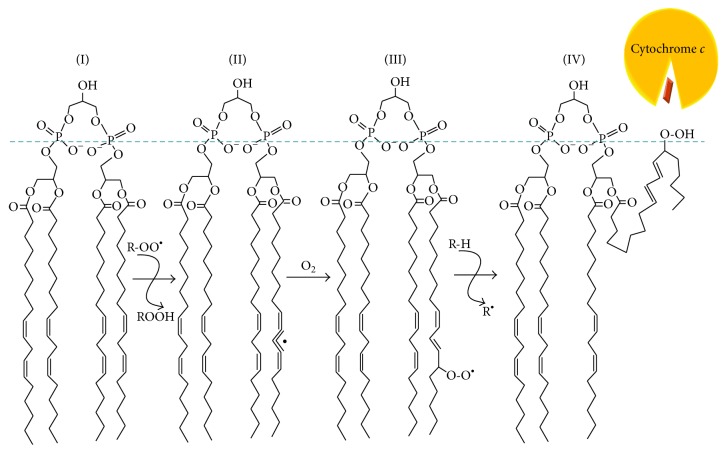
Schematic representation of the initial steps of CL peroxidation as based on [4, 10, 15–17]. The mammalian heart CL is mostly found in the form of tetralinoleoyl-CL, that is, with four polyunsaturated residues of linoleic acid (I). A bis-allylic hydrogen atom of CL may be abstracted by a peroxyl radical (ROO^*∙*^) yielding peroxide (ROOH) and a lipid-centered radical (II). The latter is involved in a fast reaction with molecular oxygen and turns into another peroxyl radical, which may consequently attack another polyunsaturated lipid in a chain process. This reaction is accompanied by rearrangement of double bonds of the fatty acid residue, which ultimately leads to the accumulation of conjugated dienes (III). Peroxide of CL exposes its nascent hydrophilic moiety at the surface of the membrane, where it may interact with cytochrome *c* and turn it into a peroxidase, thus forcing amplification of the oxidation cascade (IV).

One of the first signals of the apoptotic pathway within mitochondria is oxidized cardiolipin [5, 13, 20, 21]. A cardiolipin (CL) molecule is built of two glycerophosphate moieties, each carrying two fatty acid tails, which are connected via one more glycerol molecule (see Figure 1). Bound CL molecules are seen in the crystal structures of many membrane energy-converting complexes; see [22, 23] for reviews. Because of their four fatty acid tails, CL molecules were proposed to promote the interaction between mitochondrial respiratory enzymes [24]. Later, it was shown that CL, indeed, stabilizes supercomplexes formed of the NADH dehydrogenase (mitochondrial complex I), quinol:cytochrome *c* oxidoreductase (cytochrome *bc*_1_ complex; complex III), and cytochrome *c* oxidase (complex IV) [25–28]; a large fraction of loosely bound CL was shown to be affiliated with these supercomplexes [26–28]. Supposedly, CL molecules facilitate the integration of respiratory complexes into dynamic functional units [29, 30]. In eukaryotes, ROS are produced in mitochondria as byproducts of respiration [31, 32]; the major sources of ROS are the NADH : quinone oxidoreductase [33–35] and cytochrome *bc*_1_ complex [9, 36–40], which form CL-rich supercomplexes.

The animal variant of CL, which is closely associated with the respiratory complexes and carries polyunsaturated fatty acid chains, can be regarded as an easily “ignitable” compound. The selective peroxidation of CL in mitochondria in response to the oxidative stress was demonstrated with various experimental models [20, 41–45]. Hence, those drugs that protect CL from oxidation might postpone or prevent the cell death.

Recent years have shown notable progress in development and investigation of such drugs; see [15, 46] for reviews. It has been shown that some antioxidants can prevent both the oxidation of CL and the development of pathology. Specifically, idebenone, or 6-(10-hydroxydecyl)ubiquinone, was shown to protect the mitochondrial membrane against lipid peroxidation and improve vascular disorders—owing to strokes or experimental cerebral ischemia—as well as improve the overall brain function [47–50]. Decylubiquinone (decUQ) was shown to block ROS accumulation and prevented activation of the MPT and the cell death in the glutathione-depleted cells [51]. Treatment by propofol, a phenolic anaesthetic, was shown to protect against mitochondrial dysfunction [52].

Among others, several mitochondrially targeted antioxidants were developed; see [15, 46] for reviews. Antioxidants based on mitochondrially targeted peptides could concurrently prevent the oxidation of CL, cytochrome *c* release to the cytoplasm, and the cell death [44, 53–55].

Another way to target drugs to mitochondria is based on a covalent binding of an antioxidant to a penetrating cation. Such cations, termed “Skulachev ions” by Green [56], penetrate through biological membranes in the charged state and get distributed according to the transmembrane difference of electrical potentials [57]. Due to the ability to selectively accumulate in energized mitochondria, which are negatively charged with respect to the cell cytoplasm, such compounds can be used as “locomotives” for delivery of drugs into mitochondria [58]. Since the interior of mitochondria is by approximately 200 mV more negative than the cytosol, penetrating cations can accumulate by a factor of 1000, at least [20, 58]. A mitochondrially targeted ubiquinol analogue 10-(6′-ubiquinonyl)decyltriphenylphosphonium (MitoQ), with a triphenylphosphonium group [57] as a penetrating cation, was shown to prevent lipid peroxidation and apoptotic reactions [59, 60].

At the Lomonosov University, a series of mitochondrially targeted antioxidants comprised of a penetrating cation and an antioxidant, usually a plastoquinol group, separated by a C-10 linker, have been synthesized and tested. Such compounds were dubbed SkQ ions, that is, conjugates of penetrating Skulachev ions (Sk) and quinols (Q); these constructs were tested* in vitro* and* in vivo* [20, 61–74]. SkQs prolonged life of different organisms [68], helped animals to survive after brain and kidney ischemia [69], stroke, and heart attack [64], and decelerated the development of many age-related pathological states, such as cataract and some other eye diseases, balding, achromotrichia, lordokyphosis, and myeloid shift of the blood [20]. Owing to their ability to accumulate in mitochondria [20, 58], the SkQ ions were already efficient when added at concentrations of tens of nanomoles per kg of body weight.

Upon these studies, it has been shown that CL molecules were specifically peroxidized in mitochondria under oxidative stress, whereas other phospholipids remained more or less intact, and that 10-(6′-plastoquinonyl)decyltriphenylphosphonium (SkQ1) protected the mitochondrial CL from oxidation [20, 63, 67].

While the physiological protective effects of SkQs and some other mitochondrially targeted antioxidants are well documented (see [46] for a recent review), the mechanism of their action has remained obscure. Indeed, mitochondria possess high levels of powerful natural antioxidants, such as ubiquinols and tocopherols. The concentration of ubiquinol in the mitochondrial membrane is especially high comprising about 1% of the total lipid content [75], which corresponds to a molar concentration in the order of 10 mM. The high level of natural antioxidants in mitochondrial membranes brings us to the conundrum: why the natural stock of ubiquinol and *α*-tocopherol does not fully protect the mitochondrial CL from oxidation, whereas the artificial antioxidants, being externally added in nanomolar amounts [20], could do that?

In an attempt to answer this question, we have established a chemically defined system to quantitatively study the oxidation of CL in the membrane as a function of different antioxidants [19]. We chose pure CL membranes as an object for our kinetic studies and established production of extrusion-derived liposomes of a uniform size of about 100 nm, as confirmed by dynamic light scattering measurements and electron microscopy. The CL liposomes were impermeable for small organic molecules such as 5(6)-carboxyfluorescein [19]. To avoid protein-due effects, we initiated the oxidation of CL by an azo initiator 2,2′-azobis(4-methoxy-2,4-dimethylvaleronitrile), or MeO-AMVN [18]. The formation of primary oxidation products, conjugated dienes (see Figure 1), was followed, in real time, by measuring their accumulation at 234 nm [18, 76, 77]. The high efficiency of the hydrophobic initiator MeO-AMVN at low temperatures [18] allowed us to use low amounts of the initiator, which decreased the possible influence of the initiator on the structure of the liposomes. Our method is a direct method, and, along with polarography [10, 78], enables continuous tracing of the lipid peroxidation. However, the advantage of the spectrophotometric approach is the ability to simultaneously trace not only the accumulation of the reaction products but also the depletion of the antioxidant, which is essential for kinetic analysis of peroxidation; see Section 2 and [19] for further details on the method used.

With this experimental setup, we have already shown that the amphiphilic quinol-based antioxidants, when added to a solution of CL liposomes, were effective in protecting cardiolipin liposomes from peroxidation even in submicromolar range, with their relative effectiveness depending on the type of antioxidant group and on the nature of the membrane-targeted moiety [19].

Here, we have compared natural, hydrophobic antioxidants with artificial, amphiphilic antioxidants in a somewhat amended experimental setup. We have found that natural hydrophobic ubiquinols, when preincubated in liposomes, were as efficient as artificial amphiphilic quinol-containing antioxidants and much more efficient than chromanol-based compounds. The artificial amphiphilic antioxidants, unlike the hydrophobic ubiquinol and *α*-tocopherol, could almost fully inhibit the CL oxidation also when acting from the aqueous phase. We suggest that amphiphilic antioxidants specifically protect those CL molecules which are tightly bound within the enzymes of the respiratory chain, accounting thus for the observed therapeutic effects.

## 2. Materials and Methods

### 2.1. Chemicals

**Figure 2:**
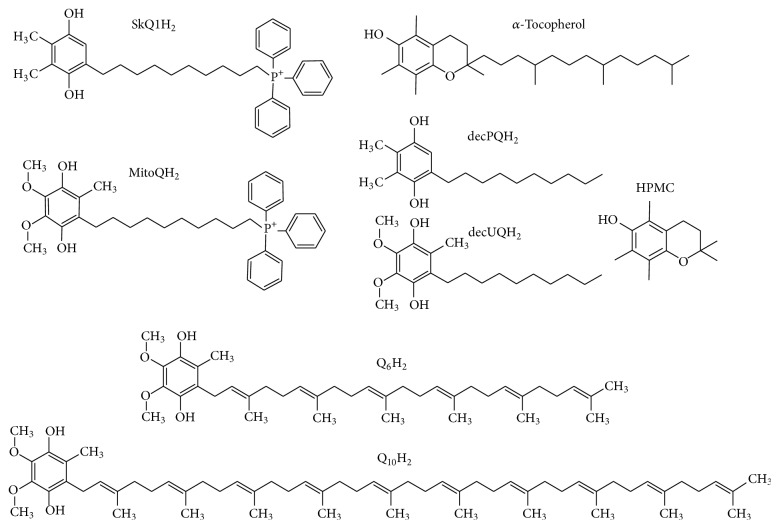
Chemical structures of the tested antioxidants. Quinone-based antioxidants are depicted in their reduced forms (i.e., as quinols).

CL from bovine heart and 1-palmitoyl-2-oleoyl-phosphatidylglycerol (POPG) were derived from Avanti Polar Lipids Inc. (Alabaster, AL, US) in the form of lyophilized powder. Yeast ubiquinone-6 was also from Avanti. Ubiquinone-10 and *α*-tocopherol were bought from Sigma-Aldrich (St. Louis, MO, US). Sodium borohydride and chloroform (99.8%) were purchased from Roth (Karlsruhe, Germany). Chemicals for buffer solutions were ordered from Sigma-Aldrich or Roth. Mini-Extruder and the porous membranes were supplied by Avanti. Azo initiator 2,2′-azobis(4-methoxy-2,4-dimethylvaleronitrile), or MeO-AMVN, was delivered by Wako Pure Chemical Industries (Osaka, Japan). HPMC was a gift of Dr. Vitaly Roginsky. Diverse triphenylphosphonium-containing quinone-based antioxidants (see Figure 2) were synthesized in their oxidized (quinone) forms as previously described [63].

### 2.2. Preparation of Liposomes

The liposomes were produced by extrusion, based on the method of Cullis and coworkers [79, 80], similarly to our previous study [19]. Liposomes were prepared by suspending lipid powder in 50 mM sodium phosphate buffer (pH 7.4) containing 100 *μ*M diethylenetriaminepentaacetic acid (buffer A) to bind possible traces of transition metals, followed by 5 minutes of vortexing to obtain a 3 mg/mL homogeneous lipid suspension. Whenever an antioxidant was incorporated into liposomes, the lipid powder was mixed with the respective antioxidant in chloroform in a glass beaker and the mixture was dried under a stream of nitrogen for at least 2 hours. Then the lipid film was hydrated by buffer A with a short series of ultrasonication followed by 3 freeze-thawing cycles to obtain lipid suspension consisting of large multilamellar vesicles (3 mg/mL). In either case large unilamellar vesicles were formed from lipid suspensions by passing them through a 100 nm pore filter using Mini-Extruder for 19 times. Kinetic studies with liposomes were performed within the day of preparation.

### 2.3. Converting Quinones to Quinols

Reduction of quinones to corresponding quinols was performed by adding a small amount (2-3 mg) of dry sodium borohydride to 1-2 mM quinone solution in ethanol. The reaction was complete within a minute of intensive mixing. The excess of reductant was removed by a small volume of fuming HCl followed by at least two centrifugations at 15800 g (5 minutes each) to remove the sodium borate pellet. Reduced quinones were stored at −80°C until use. The final quinol concentration was determined from the absorbance maximum at 290 nm. The molar absorptivity coefficients used for ubiquinol-6, ubiquinol-10, plastoquinol-based antioxidants (SkQ1H_2_ and decPQH_2_), and amphiphilic ubiquinol-based antioxidants (MitoQH_2_ and decUQH_2_) were 4890, 3940 M^−1 ^cm^−1^ [81], 3540 M^−1 ^cm^−1^ [82], and 4140 M^−1 ^cm^−1^ [83], respectively. Under acidic conditions the reduced state of quinols was preserved for months. The list of all antioxidants in their reduced forms is presented in Figure 2.

### 2.4. Oxidation of HPMC

The oxidized form of HPMC (the respective quinone or other products) was achieved by overnight incubation of 5 *μ*M HPMC with 10 *μ*M of CuCl_2_ at 40°C in 50 mM sodium phosphate pH-buffer, pH 7.4.

### 2.5. Experimental Setup for Lipid Peroxidation Assays

**Figure 3:**
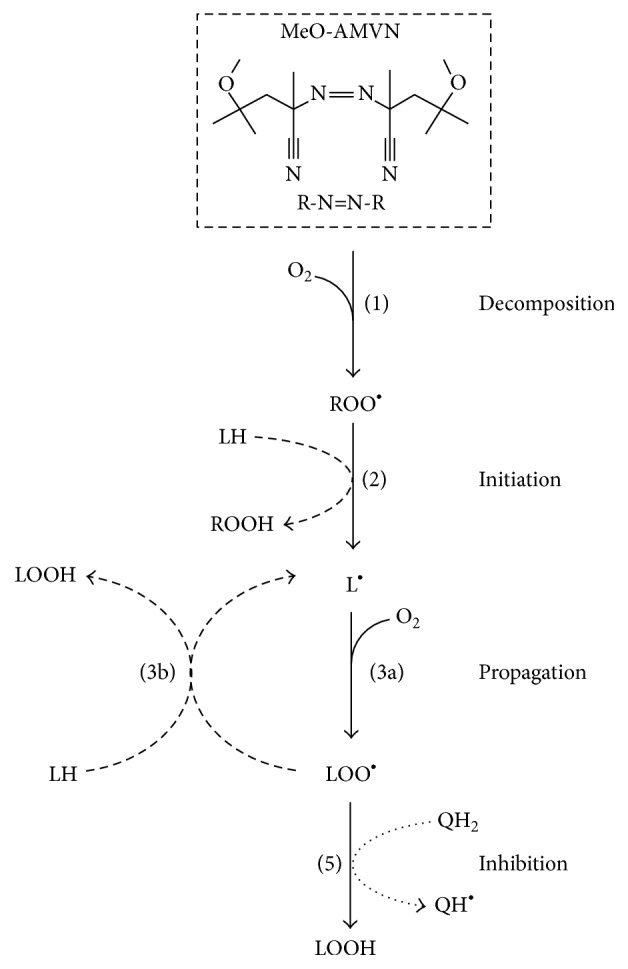
Scheme of a chain radical reaction in a system containing an azo initiator MeO-AMVN, CL molecules, and a chain-breaking inhibitor of quinol nature (based on [4, 10, 15–18]). The main stages of the process are indicated by numbers corresponding to the respective reactions, as described in Section 2. The radical products of the initiator decomposition (1) ROO^*∙*^ initiate the oxidative cycle via formation of an alkyl (L^*∙*^) radical (2). The reaction propagates ((3a) and (3b)) as this alkyl radical consumes molecular oxygen to form peroxyl (LOO^*∙*^) radical (3a), which abstracts a hydrogen atom from another lipid molecule (LH) to yield another L^*∙*^ radical (3b). An inhibitor, such as quinol (QH_2_), may break the reaction chain by quenching a LOO^*∙*^ radical (5).

As shown in Figure 3, the lipid peroxidation was initiated by an azo initiator, based on approaches described in [18, 76, 77], similarly as in our previous study [19].

Following Noguchi et al. [18], we launched the peroxidation of CL by adding the peroxyl radical azo initiator MeO-AMVN. This substance spontaneously decomposes, with a rate that is constant at a given temperature, into two carbon-centered radicals, which promptly become peroxyl radicals in an oxygen-rich medium (Figure 3).

*Decomposition*:

(1) R-N=N-R⟶2R∙+N2R∙+O2⟶ROO∙

The peroxyl radical (ROO^*∙*^) may abstract a bis-allylic hydrogen atom from a polyunsaturated lipid molecule, thus initiating the chain.

*Initiation:*

(2) $$\begin{array}{c} ROO^{∙}+LH⟶L^{∙}+ROOH \end{array}$$

The resulting carbon-centered lipid radical L^*∙*^ will further react with oxygen molecules to give a peroxyl radical of a lipid (LOO^*∙*^). This radical, in turn, may attack other polyunsaturated lipids, thereby accounting for the propagation of the oxidation chain.

*Propagation*:

(3a) L∙+O2⟶LOO∙

(3b) LOO∙+LH⟶L∙+LOOH

The process continues until two radicals terminate in a bimolecular reaction.

*Termination:*

(4) $$\begin{array}{c} 2\;LOO^{∙}⟶\text{nonradical products} \end{array}$$

These first stages of polyunsaturated fatty acid peroxidation lead to formation of monohydroperoxide accompanied by the rearrangement of double bonds to form conjugated dienes [4, 84, 85] (see Figures 1 and 3). Conjugated dienes have a broad absorption band with a maximum at 234 nm, which can be monitored spectroscopically. If the majority of polyunsaturated fatty acid residues represent linoleic acid, as in the case of bovine heart CL, the number of peroxides formed would be stoichiometrically equal to the number of conjugated dienes formed [10, 86]. Since we used a mild initiator only capable of producing peroxyl radicals, and the medium was purged from transition metal ions with a chelator, the reaction would halt at the first diene-peroxide stage. This method is both direct, as it quantifies the primary reaction product [10], and precise in terms of the signal to noise ratio.

Chain-breaking antioxidants, such as tocopherols or quinols, react with peroxyl radicals much faster than the bis-allylic hydrogen atom of a fatty acid, and thereby can prevent the chain propagation.

*Inhibition:*

(5) $$\begin{array}{c} QH_{2}+LOO^{∙}⟶QH^{∙}+LOOH \end{array}$$

The product of such reaction, a semiquinone (QH^∙^) or an *α*-tocopheroxyl (*α*-toc^*∙*^) radical, can either quench another lipid radical species

(6) $$\begin{array}{c} QH^{∙}+LOO^{∙}⟶Q+LOOH \end{array}$$

or be quenched by another radical species of the same antioxidant. In case of semiquinone, this will partly regenerate the initial quinol molecule via disproportionation:

(7) $$\begin{array}{c} 2\;QH^{∙}⟶Q+QH_{2} \end{array}$$

The products of collision of two *α*-tocopheroxyl radicals may give rise to different substances; see [10].

In the ideal case, when an antioxidant and its oxidation-derived radical form both participate in the quenching of lipid peroxyl radicals, the stoichiometry of the inhibition would correspond to two quenched radicals per one consumed antioxidant molecule. In reality, the stoichiometry is usually lower because of losses. For instance, a semiquinone radical can be oxidized by molecular oxygen yielding a superoxide anion and a fully oxidized quinone molecule as products:

(8) $$\begin{array}{c} QH^{∙}+O_{2}⟶Q+{O_{2}}_{}^{∙-}+H^{+} \end{array}$$

Ultimately, if the extent of the autooxidation is very high, the stoichiometry of the quenching of peroxyl radicals by quinols may be even close to zero [78, 87]. Unlike quinols, both *α*-tocopherol and its *α*-tocopheroxyl radical are practically nonreactive towards oxygen [88], which implies that *α*-tocopherol and its analogues have an inhibitory stoichiometry of two, being often viewed as reference antioxidants.

Oxidation assays were performed in buffer A at final CL concentration of 100 *μ*M. The sample was incubated in a 3 mL quartz cuvette inside a UV-2450 spectrophotometer (Shimadzu, Tokyo, Japan) equipped with a Peltier element at 40°C. The experiment was initiated by addition of 50 *μ*M MeO-AMVN.

**Figure 4:**
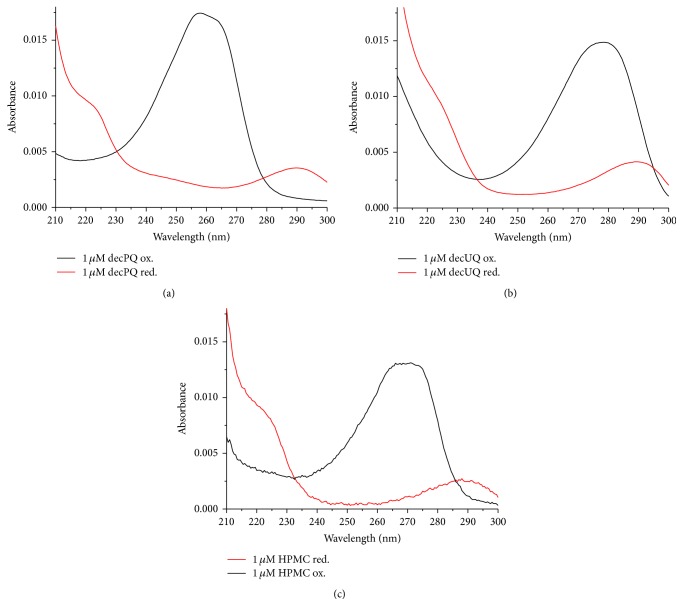
Spectra of oxidized (black line) and reduced (red line) forms of (a) decPQ, (b) decUQ, and (c) HPMC (see text). The spectra were normalized to 1 *μ*M of the substance to assess the impact of oxidation of these compounds into total absorbance changes during the experiment. All the three redox pairs have isosbestic points at 230–240 nm suggesting a minimal interference with the robust signal of conjugated dienes. The ubiquinone/ubiquinol pair also has an “oxidized* minus* reduced” difference absorption maximum at ~275 nm, which does not strongly overlap with the spectrum of conjugated dienes.

Oxidation of CL was monitored by changes in absorbance at 234 nm which corresponds to formation of conjugated dienes [18, 89, 90]. The value taken for the molar absorptivity of conjugated dienes was 27400 M^−1 ^cm^−1^. Antioxidants themselves also absorbed in the UV range; however their difference “oxidized minus reduced” spectra had isosbestic points close to 234 nm (Figure 4) and therefore could not notably contribute to the absorbance changes as monitored at 234 nm. Spectra at the wavelengths of 210–300 nm were recorded every 5 minutes against a reference cuvette containing all the same components, except for MeO-AMVN. The noise produced by the setup was always in the range of 0.002 absorbance units at 230–300 nm. Antioxidants were introduced to both cuvettes either initially, when incorporated into CL liposomes, or 30 minutes after the start of the experiment, when incorporated into POPG liposomes, or added as ethanol solutions. The POPG stock suspension used for antioxidant delivery usually contained 20 or 100 *μ*M of antioxidant incorporated into 4 mM of POPG.

### 2.6. Statistical Analysis of the Data

The statistical analysis was performed with MATLAB software. The data for pure CL oxidation were obtained from five experiments when no antioxidants were incorporated. The data for natural and amphiphilic antioxidants were derived from the plateau region by averaging three runs for each antioxidant incorporated into CL or POPG liposomes, and two runs for MitoQH_2_. The data for HPMC were averaged from five separate runs including experiments with CL incorporation, POPG incorporation, and external addition to CL liposomes in ethanol, as described in our previous publication [19]. The duration of inhibition phase for each antioxidant was determined as a time point of intersection of the lines corresponding to the average kinetics of inhibited and noninhibited oxidation.

## 3. Results

### 3.1. Preincorporation of Antioxidants into Cardiolipin Liposomes

**Figure 5:**
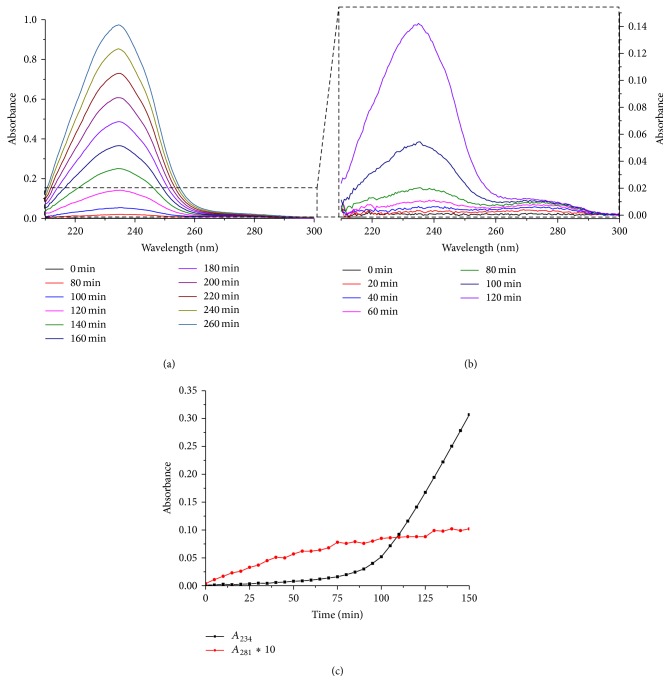
Prevention of the MeO-AMVN-induced oxidation in CL liposomes by 1 *μ*M Q_10_H_2_. Conditions: pH-buffer A (see Section 2), 100 *μ*M CL, 50 *μ*M MeO-AMVN, and 40°C. (a, b) Spectral changes of the liposome suspension over the whole time range (a) and during the induction phase (b). (c) Kinetics of absorbance changes at 234 nm (accumulation of conjugated dienes) and at 281 nm (“oxidized* minus* reduced” absorption difference for Q_10_, cf with Figure 4(b)) show that lipid peroxidation did not start before the antioxidant Q_10_H_2_ was exhausted.

In the first set of experiments, we incorporated the antioxidants into the hydrophobic lipid phase as described in Materials and Methods. Figures 5(a) and 5(b) show the UV absorbance changes during the MeO-AMVN-induced CL peroxidation in the sample that contained 1 *μ*M Q_10_H_2_ incorporated into 100 *μ*M of liposomal CL. The most remarkable changes occurred around 234 nm and 281 nm (Figure 5(c)), which correspond to the absorption maxima of conjugated dienes and “oxidized* minus* reduced” ubiquinone (Figure 4), respectively. First, in response to the addition of MeO-AMVN, the fast formation of ubiquinone was observed (Figure 5(c)). Concurrently, a small dip was observed at 290 nm (Figure 5(b)), which reflected the disappearance of ubiquinol. Only after ubiquinol got exhausted in approximately 100 minutes, a broad peak of conjugated dienes with a maximum at 234 nm appeared and started to grow (Figure 5(c)). The start of absorbance rise at 234 nm correlated well with a plateau in the *A*_281_ kinetics (at a time point of ~100 minutes). Thus, the protective action of Q_10_H_2_ was not compromised until the full expenditure of the antioxidant.

**Figure 6 fig6:**
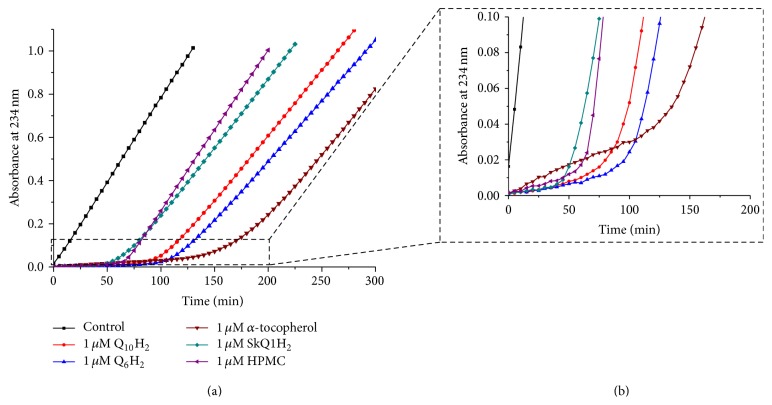
Prevention of the MeO-AMVN-induced oxidation of CL liposomes by incorporated natural antioxidants, SkQ1H_2_ and HPMC. Conditions: pH-buffer A (see Section 2), 100 *μ*M CL, 50 *μ*M MeO-AMVN, and 40°C. (a, b) Kinetics of absorbance changes at 234 nm in the presence of 1 *μ*M of tested incorporated antioxidants and in their absence (black line) over the whole time range (a) and only during the induction phase (b).

Figures 6(a) and 6(b) show the kinetics of CL oxidation in the presence of three natural antioxidants, namely, Q_10_H_2_, Q_6_H_2_, and *α*-tocopherol, as well as artificial antioxidants, amphiphilic plastoquinol-based SkQ1H_2_, and 6-hydroxy-2,2,5,7,8-pentamethylbenzochroman (HPMC), a synthetic *α*-tocopherol analogue lacking the hydrophobic tail (see Figure 2 for the structures). The initial slopes of the kinetic curves (see the insert in Figure 6(b)) correspond to the effectiveness of the tested substances as antioxidants. Apparently, the quinol-based antioxidants almost fully protected CL from oxidation, unlike *α*-tocopherol, in the presence of which a notable oxidation of CL still took place. All the quinols, both natural and synthetic, demonstrated equal antioxidant activity, but the duration of the inhibitory action was almost twice longer for Q_10_H_2_ and Q_6_H_2_ as compared with SkQ1H_2_. The duration of the antioxidant activity of *α*-tocopherol was the longest. The antioxidant potency of HPMC was lower than that of the quinols and slightly higher than that of *α*-tocopherol, although the duration of the induction period was much shorter.

**Figure 7 fig7:**
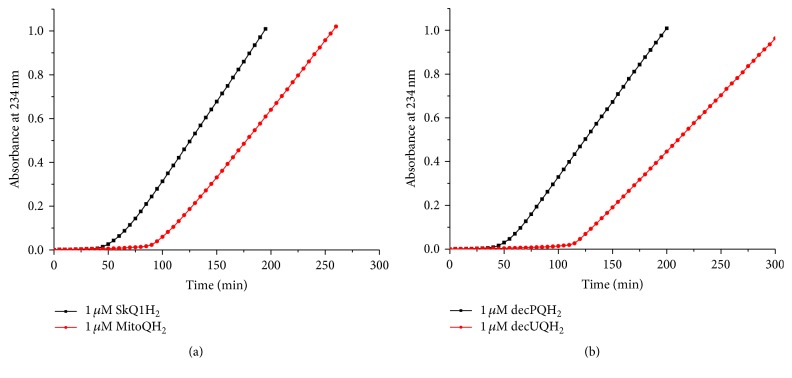
Prevention of the MeO-AMVN-induced oxidation in CL liposomes by incorporated amphiphilic, quinol-based antioxidants. Conditions: pH-buffer A (see Section 2), 100 *μ*M CL, 50 *μ*M MeO-AMVN, and 40°C. (a, b) Absorbance changes at 234 nm in the presence of decyl-containing antioxidants (a) and decyltriphenylphosphonium-containing antioxidants (b).

We have also examined other amphiphilic analogues of ubiquinol (see Figure 2 for the structures) using the same experimental setup. Namely, we tested the aforementioned MitoQH_2_, a compound similar to SkQ1H_2_ but with an ubiquinol group instead of the plastoquinol moiety of SkQ1H_2_ (Figure 7(a)), and amphiphilic short-tail quinols lacking penetrating cation moieties, decPQH_2_ and decUQH_2_ (Figure 7(b)). In both cases, the antioxidant efficiency of all tested compounds was high, but the duration of protection was almost twice as long in the case of ubiquinol-based antioxidants.

**Figure 8 fig8:**
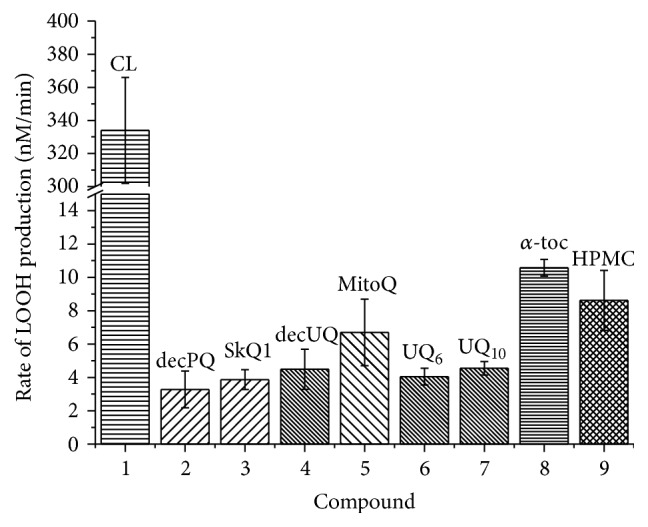
The rate of oxidation of liposomal CL in the presence of various antioxidants (data from Figures 5 [Fig fig6]–7 and 10; see also Table 1). 1 *μ*M of hydrophobic or amphiphilic antioxidants was added to 100 *μ*M of CL liposomes in the presence of 50 *μ*M of MeO-AMVN (40°C). The mean values are represented as bars, and the standard deviations are shown as ticks (see Section 2).

The quantitative comparison of different antioxidants incorporated into CL or POPG liposomes (see also Section 3.2) is given in Figure 8 and Table 1. The data correlate with our previous study where the amphiphilic quinol-based antioxidants were potent inhibitors of CL peroxidation when added externally as ethanol solutions [19].

### 3.2. Preincorporation of Antioxidants into Nonoxidizable Liposomes

**Figure 9 fig9:**
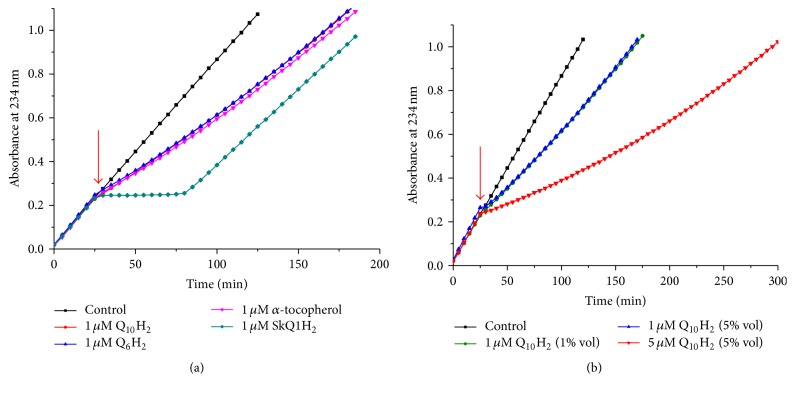
Prevention of MeO-AMVN-induced oxidation of CL liposomes by antioxidants added externally within POPG liposomes. Peroxidation was started by addition of 50 *μ*M MeO-AMVN to 100 *μ*M CL liposomes at 40°C in buffer A. Antioxidant-containing POPG liposomes were added 30 minutes after the start of the experiment, as indicated by arrows. (a) Comparison of SkQ1H_2_ with natural hydrophobic antioxidants. In each case the final concentrations were 1 *μ*M of antioxidants and 40 *μ*M of POPG. (b) Radical quenching efficiency of ubiquinol-10 as function of its concentration and the extent of dilution. The amount of added POPG stock suspension was 1% (30 *μ*L) or 5% (150 *μ*L) of the total system volume, which accounts for 40 or 200 *μ*M POPG, respectively.

The data in Figures 6 [Fig fig7]–8 and Table 1 indicate that all tested quinol-based antioxidants efficiently protected liposomal CL from oxidation; still with this approach we could not compare the kinetics of CL oxidation in the absence and in the presence of an antioxidant, respectively, because antioxidants were added already upon the preparation of liposomes. In our previous study [19], we added antioxidants as an ethanol solution after liposomes underwent MeO-AMVN-initiated peroxidation for 30 minutes. This approach, however, is of little use with hydrophobic, water-insoluble antioxidants. Therefore, we combined preincorporation of hydrophobic antioxidants into lipid bilayer with an external way of their supplementation to CL liposomes. For this purpose, antioxidants were incorporated into liposomes made of an inert, nonoxidizable lipid 1-palmitoyl-2-oleoyl-phosphatidylglycerol (POPG), and these antioxidant-loaded liposomes were added to a suspension of CL liposomes that underwent MeO-AMVN-driven peroxidation for 30 minutes (Figures 9(a) and 9(b)). In this setup we tested the antioxidant potency of Q_10_H_2_, Q_6_H_2_, and *α*-tocopherol, as well as of SkQ1H_2_ (Figure 9(a)). The generation of conjugated dienes was essentially blocked by the addition of liposomes that contained SkQ1H_2_; the duration of the lag period was similar to that observed earlier when the same antioxidant was added in an ethanol solution (~60 min, see [19]). Natural hydrophobic antioxidants, being added, within liposomes, to the same final concentration of 1 *μ*M, retarded the oxidation of CL only slightly, and to the same extent. To understand the reason of this retardation, we have studied the impact of Q_10_H_2_ in some more detail (Figure 9(b)). The effect of Q_10_H_2_ was found to be concentration-dependent, as 5 *μ*M of Q_10_H_2_ retarded the peroxidation more efficiently than 1 *μ*M of Q_10_H_2_. In contrast, the efficiency was independent of the lipid : antioxidant ratio in the added POPG suspension. 1 *μ*M of Q_10_H_2_ introduced with 200 *μ*M POPG was almost as efficient as 1 *μ*M of Q_10_H_2_ added within 40 *μ*M POPG (Figure 9(b)). Apparently, only the final concentration of Q_10_H_2_ determined the efficiency, but not the amount of externally added POPG. It is noteworthy that in both experiments (Figures 9(a) and 9(b)) the addition of POPG liposomes without antioxidants (the control sample) did not affect the rate of CL peroxidation.

**Figure 10:**
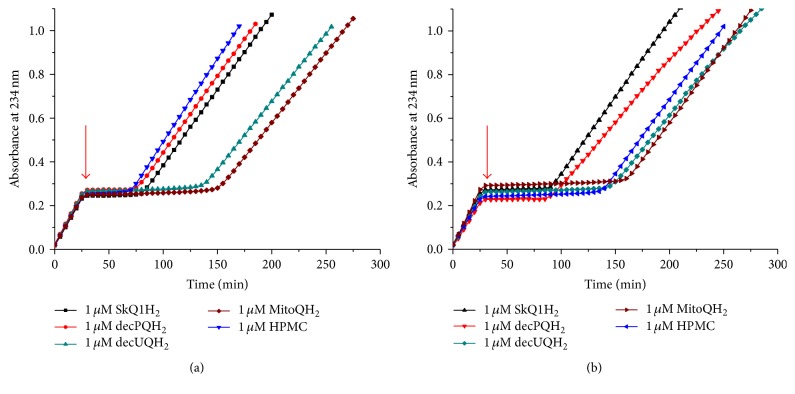
Prevention of MeO-AMVN-induced oxidation in CL liposomes by various antioxidants added externally within POPG liposomes (a) or as ethanol solutions (b). Peroxidation was started by addition of 50 *μ*M MeO-AMVN to 100 *μ*M CL liposomes at 40°C in buffer A. The moments of addition of the antioxidants (30 minutes after the start of the experiment) are marked by arrows. (a) Effects of 1 *μ*M SkQ1H_2_, 1 *μ*M decPQH_2_, 1 *μ*M decUQH_2_, 1 *μ*M MitoQH_2_, or 1 *μ*M HPMC added in POPG liposomes to CL liposomes within 40 *μ*M POPG (1% volume). (b) Effects of 1 *μ*M SkQ1H_2_, 1 *μ*M decPQH_2_, 1 *μ*M decUQH_2_, 1 *μ*M MitoQH_2_, or 1 *μ*M HPMC added as ethanol solutions, see also [19].

With externally added liposomes we delivered also some further analogues of natural antioxidants, namely, HPMC, MitoQH_2_, decPQH_2_, and decUQH_2_, to CL liposomes (Figure 10(a)). All these amphiphilic antioxidants were able to diffuse between liposomes via the water phase and prevent the oxidation of CL. Similarly to the CL-incorporated antioxidants (Figures 6 and 7) and the antioxidants introduced as ethanol solutions [19], the ubiquinol-based antioxidants (decUQH_2_, MitoQH_2_) showed an approximately twice as long inhibitory period compared to plastoquinol-based antioxidants (decPQH_2_, SkQ1H_2_). Here, HPMC was again barely able to incorporate into the POPG liposomes upon their preparation accounting for the shortest lag period, as compared to other tested antioxidants (Figure 10(a)).  Figure 10(b) shows the antioxidant action of compounds that were delivered to the suspension of CL liposomes being dissolved in ethanol, using the approach from our previous study [19]. In this case, HPMC provided a much longer protection with a stoichiometry close to two trapped radicals per one antioxidant molecule.

## 4. Discussion

The present work complements our previous study of quenching the peroxidation of CL liposomes by amphiphilic antioxidants dissolved in ethanol [19]. Here, we tested natural hydrophobic antioxidants such as *α*-tocopherol and ubiquinols, by preincorporating them into the hydrophobic environment of liposomes.

The relative antioxidant efficiency of ubiquinols and tocopherols was earlier assessed in numerous natural and artificial systems and was shown to depend on the nature of the system tested [16, 91–100].

In homogeneous systems, such as hexane [91, 92] or acetonitrile [92], *α*-tocopherol was shown to be more efficient than ubiquinol-10 in protecting fatty acid esters from oxidation.

In heterogeneous systems containing polar/nonpolar interfaces, the affinity of both the antioxidant and substrate—the lipid peroxyl radical—to the interfaces could affect the kinetics of radical quenching. In simplest biphasic micellar systems the antioxidant potencies of quinols and *α*-tocopherol were shown to be comparable [78, 92, 101]. In the case of low density lipoprotein (LDL) particles, the carriers of fatty acids in the blood, the literature data on relative antioxidant capacities of ubiquinol and *α*-tocopherol indicate a higher protective effect of ubiquinol [93, 97].

Unilamellar liposomes represent a good model for quantitative studying of antioxidants [102]. While liposomes share many traits with physiological systems, their oxidation kinetics can be described by the law of mass action [103, 104]. However, liposomal phospholipid membranes are even more complex than micelles or LDL particles because of the complex nature of their interfaces formed of polar lipid heads that are connected to the hydrophobic tails via glycerol moieties and phosphate groups. The redox active moieties of tocopherols [105] and natural ubiquinols [106–108] are expected to dwell on the level of these phosphate groups. Therefore, interactions of antioxidants with these membrane embedded polar groups may additionally affect the antioxidant efficiency and other properties of tocopherols and ubiquinols. Furthermore, the exact position of the polar “phosphate” layer might depend on the nature of the phospholipid; molecular dynamics simulations showed that the phosphate to phosphate distances increased from about 3.8 nm to about 4.7 nm when the CL content of a POPC membrane changed from 0% to 100% [109]. Therefore, the relative antioxidant efficiency of tocopherols and ubiquinols, as well as their other properties, may depend on the nature of phospholipids and should be determined experimentally for different lipid membranes.

According to the here presented data, all the tested quinols were equally highly effective in protecting CL from peroxidation, as indicated by negligible slopes of absorbance increase at 234 nm in their presence; see Figures 5 [Fig fig6] [Fig fig7]–8 and 10, as well as the Table 1. In agreement with previous observations [19], even minute, residual amounts of the antioxidant were sufficient to protect CL from peroxidation. At the same time, *α*-tocopherol and its hydrophilic analogue HPMC were less effective (Figures 6 and 10). These results were rather unexpected, since some of the previous data on concurrent measurements of antioxidant activities of *α*-tocopherol and ubiquinols in lipid bilayers were interpreted as indicating *α*-tocopherol to be either an equally potent [94], or even a better [91] antioxidant than ubiquinol. In these experiments, where both *α*-tocopherol and ubiquinol-10 were present as antioxidants, ubiquinol was consumed before *α*-tocopherol [91, 92, 94, 97, 110, 111]. Building on the previously observed kinetic superiority of *α*-tocopherol over ubiquinol in homogenous model systems [16, 91, 92] and the observed ability of ubiquinol to recover the *α*-tocopheroxyl radical [92, 112], it was generally implied that the faster expenditure of ubiquinol was only apparent, being due to repletion of the promptly oxidized *α*-tocopherol by ubiquinol; see [113, 114] for reviews. Our kinetically resolved data clarify this conundrum, at least, for CL membranes. It follows from our data that all the tested quinol-based antioxidants distinctly outperformed *α*-tocopherol and its hydrophilic analogue HPMC. The faster oxidation of ubiquinol in the presence of *α*-tocopherol in the earlier experiments with liposomes [91, 92, 94, 97, 110, 111] was, most likely, due to the genuinely faster interaction of ubiquinol with radical species. To what extent our results could be extrapolated to multicomponent mitochondrial membranes, where the potency of different antioxidants is constrained by the rate of their “recycling” by components of the electron transfer chain [63, 110, 111], is yet to be established.

The radical-scavenging stoichiometry for *α*-tocopherol equals two trapped peroxyl radicals per antioxidant molecule under most of the experimental conditions [10]; the same should be true for its hydrophilic analogue HPMC. Indeed, when HPMC was added as ethanol solution (Figure 10(b)) the duration of its action was almost as long as that of ubiquinol-based antioxidants. The shorter duration of action of HPMC upon its addition into liposomes (Figures 6 and 10(a)) might reflect the loss of hydrophilic HPMC upon the sample preparation.

From our data, we could approximately estimate the chain propagation length of CL peroxidation in the membrane, that is, the number of peroxidized fatty acid chains per one radical as generated upon thermal decomposition of MeO-AMVN (see Figure 3). Typically, this value could be obtained from an experiment where the oxidation is inhibited by an antioxidant with known stoichiometry, for example, *α*-tocopherol, for which the number of trapped radicals has been estimated as two [10]. During the lag phase caused by the addition of a potent antioxidant, all the radicals generated are assumed to be eventually quenched, so that the total number of generated radicals should match the concentration of the added antioxidant.

In our experiments, *α*-tocopherol, a reference antioxidant for such calculations, could not block the oxidation of CL completely, unlike quinols.

According to the data in Figure 6, the duration of plateau due to the oxidation of 1 *μ*M *α*-tocopherol was about 170 minutes. Assuming that all radicals produced by the MeO-AMVN decomposition were finally trapped by *α*-tocopherol, and two radicals were taken per one *α*-tocopherol molecule, the rate of radical generation within this system would be 1 *μ*M/170 min × 2 = 11.8 nM/min. At the end of plateau the absorbance at 234 nm (*A*_234_) reached approx. 0.04; this corresponded to ~1.5 *μ*M of peroxidized lipid tails (given that the molar extinction of conjugated dienes is about 27400 M^−1 ^cm^−1^); that is, the oxidation rates of *α*-tocopherol and CL coincided under such conditions. Thus, *α*-tocopherol managed to trap directly about half of the radicals that would otherwise attack CL molecules in the absence of the antioxidant.

To calculate the rate of lipid oxidation, *R*_ox_, as caused by this radical flux, we calculated the rate of lipid oxidation without inhibitors (the first 25 minutes of the experiment in Figure 10(a)). This value may be derived from the accumulation of conjugated dienes as followed at 234 nm, assuming that the molar extinction coefficient for conjugated dienes is 27400 M^−1 ^cm^−1^:

(9) Roxclipidt=A23425 min−A2340 min27400×25 min=3.3×10−7 M×min−1.

Hence, the rate of uninhibited CL oxidation was ~330 nM/min, resulting in a chain propagation length of the CL peroxidation cycle of approximately 28. In the case of natural ubiquinols as antioxidants the chain propagation length was less than 0.4 (see Table 1), which indicates that the quenching of radicals by the antioxidant (see (5)) was approx. three times faster than the formation of a fatty acid peroxide (see (3a)). Hence, almost all radicals were quenched before getting the opportunity to oxidize a fatty acid, so that quinols essentially prevented the propagation of lipid oxidation. In the presence of *α*-tocopherol, the chain propagation length was about 0.9, which roughly corresponds to the quenching of only each second radical (see also above).

The duration of the inhibitory effect, as tested here and in the previous study [19], was, in both cases, related to the nature of the antioxidant moiety, decreasing in the series *α*-tocopherol ~ ubiquinol > plastoquinol, independently of the rest of the structure of the antioxidant; compare Figures 5 [Fig fig6]–7, 9, and 10, Table 1, and [19]. Hence, although all the quinols got fully oxidized at the end of the lag phase, the duration of the inhibitory action of plastoquinols (this work) and several other quinols [19] was approximately two times shorter than the respective duration for ubiquinols, which, in turn, was almost as long as the duration of the protective effect of *α*-tocopherol. Since *α*-tocopherol is known to quench stoichiometrically two peroxyl radicals per molecule [10, 16, 92], the number of quenched radicals could be estimated as slightly less than two for ubiquinols and as about one for other tested quinols.

As discussed elsewhere [10, 115, 116], the decrease in the number of quenched radicals in the case of quinols might be due to the competition of radical species with oxygen for the semiquinones of the antioxidant (see (8)). Most likely, the shorter oxidation time of quinols as compared to *α*-tocopherol was due to the partial oxidation of semiquinones by the oxygen; see the discussion below and [115, 116]. The slower expenditure of methoxy-substituted ubiquinols, as compared to other quinols, was earlier noted in experiments where oxidation of styrene and methyl linoleate in micelles was induced by a water-soluble azo initiator [78, 87]; the difference was attributed to the slower oxidation of methoxy-substituted semiquinones by oxygen (see (8)) as compared to other semiquinones.

The slower oxidation of methoxy-substituted ubisemiquinones by oxygen (Figures 6, 7, 9, and 10) may have several reasons. Some of the available estimates of the standard redox potential at neutral pH (*E*_*o*_^7^) for the ubiquinone/ubisemiquinone redox pair for protic solvents, such as methanol or water, are higher (up to 90 mV [117]) than the estimates for *E*_*o*_^7^ for the oxygen/superoxide pair under the same conditions (approx. −150 mV) [116, 118]. Conversely, the available estimates for *E*_*o*_^7^ of the plastoquinone/plastosemiquinone pair are lower, for example, −165 mV [115]. With these numbers taken at a face value, the equilibrium of the reaction

(10) $$\begin{array}{c} O_{2}+SQ⟷{O_{2}}_{}^{∙-}+Q \end{array}$$

should be shifted to the left in case of ubiquinone and to the right in case of plastoquinone, in agreement with our observations and also the earlier reports on the long life of pulse-generated ubiquinone radicals in the presence of oxygen [117, 119].

Still, the case might be not so simple. In fact, the potentials of redox pairs that involve radical states strongly depend on the environment and the measurement conditions. Generally, any interaction that would selectively stabilize the polar semiquinone molecule should increase the respective *E*_*o*_^7^ value [37, 115, 120]. Therefore, for semiquinone-involving redox pairs the very term “standard redox potential” (*E*_*o*_) is meaningful only in relation to particular homogeneous solvents such as water, methanol, or *n*-hexane [115]. For micelles or phospholipid bilayers it is more appropriate to use a “working” term “midpoint redox potential” (*E*_*m*_) for the efficient redox potential; a term being applicable only to the given system and only under given conditions. No systematic determination of midpoint potentials of quinones in CL bilayers has been done so far. Therefore, the thermodynamics of quinol oxidation in CL membranes awaits its investigators. In addition, the methoxy groups of ubisemiquinones can kinetically hamper the interaction with oxygen, as discussed in more detail in [19]. Particularly, a methoxy group might render the addition of dioxygen molecule to the benzene ring of the semiquinone, which seems to precede the subsequent elimination of superoxide [121].

Hence, as it follows from our data, ubisemiquinone radicals were much less susceptible to oxygen than radicals of other semiquinones when studied within a CL bilayer, which modeled the mitochondrial environment of respiratory complexes. The relative contributions of thermodynamic and kinetic factors in rendering the oxidation of ubisemiquinones by oxygen deserve further clarification. The physiological and evolutionary implications of the poor oxidizability of ubisemiquinones by oxygen are discussed elsewhere [122].

As it follows from Figures 5 [Fig fig6] [Fig fig7]–8 and 10 as well as from Table 1, the quinols of the same type (ubiquinols, plastoquinols) but differing in the side chain structure (with decyl, decyltriphenylphosphonium, or prenyl tails) protected CL liposomes from oxidation with similar efficiency. This is also true for *α*-tocopherol and its hydrophilic analogue HPMC (see Figures 6 and 10). This finding correlates with the previous studies where the antioxidants bearing the same active group were equally efficient in protecting liposomes made of two-tail lipids [76, 123].

In experiments with two types of liposomes, namely, oxidizable CL liposomes and antioxidant-containing, nonoxidizable POPG liposomes (Figure 9), all the three tested natural hydrophobic antioxidants (Q_6_H_2_, Q_10_H_2_, and *α*-tocopherol), being much less efficient than SkQ1H_2_, slowed down the peroxidation of CL to the same extent (Figure 9(a)). Although we used a hydrophobic azo initiator MeO-AMVN with the oil/water distribution coefficient of about 1000 [124], the prevalence of the aqueous phase in our system over the lipid phase and the more hydrophilic nature of the MeO-AMVN-derived peroxyl radicals as compared to MeO-AMVN suggest that lipid peroxidation would be almost exclusively induced from the water phase, which agrees with the fact that the MeO-AMVN radicals can be removed by size exclusion gel filtration [124]. In this case, the extent of radical quenching would not depend on the amount of nonoxidizable lipid. Indeed, a fivefold increase in the amount of nonoxidizable lipid did not affect the rate of CL oxidation (Figure 9(b)).

The rate of CL peroxidation decreased, however, when the total amount of incorporated ubiquinol-10 was increased; see Figure 9(b). It is unlikely that highly hydrophobic antioxidants could escape the POPG membrane and relocate into the CL liposomes to protect them. The exchange of *α*-tocopherol between liposomes was estimated to be in the order of hours [125, 126]; for more hydrophobic Q_6_H_2_ and Q_10_H_2_ the exchange time should be even longer. In all likelihood, the antioxidants in POPG liposomes “competed” with CL molecules for the water-dissolved MeO-AMVN radicals. In Figure 9, 1 *μ*M of hydrophobic antioxidant in POPG liposomes slowed down the oxidation of CL liposomes only by a factor of 3, although the rate constant of radical quenching by an antioxidant (about 3 × 10^5^ M^−1^ s^−1^) is 5000 times larger than the rate constant of hydrogen abstraction from polyunsaturated linoleate acid (60 M^−1^ s^−1^; see [122] and the references therein). One has to consider, however, that 1 *μ*M of antioxidant competed with 100 *μ*M of CL. Taking into account four polyunsaturated tails per 1 CL molecule and the chain reaction length of 28 (see above), one gets acceptable correspondence with the experimental data. In the framework of the suggested rational, one would expect that a 5-fold increase in the amount of antioxidant would decrease the rate of lipid peroxidation also fivefold. We have observed only a threefold drop in the rate (Figure 9(b)); the reason for this minor discrepancy with the expectations should be yet clarified.

While ubiquinols were more efficient than *α*-tocopherol in experiments where antioxidants were added to CL upon preparation of liposomes, the efficiency of natural ubiquinols and *α*-tocopherol was similarly low when they were incorporated in the POPG liposomes (Figure 9(a)). In the latter case, the rate of CL oxidation should slow proportionally to the fraction of the MeO-AMVN radicals that were quenched in the hydrophobic phase of the antioxidant-containing POPG liposomes. Under the assumption that the fraction of MeO-AMVN radicals that got into the hydrophobic phase of POPG liposomes was independent of the nature of the antioxidant, the data obtained indicate that almost all MeO-AMVN radicals that got into the hydrophobic phase of the POPG liposomes could be quenched, independently of the nature of incorporated hydrophobic antioxidant.

In contrast to hydrophobic antioxidants, the amphiphilic antioxidants, when added with POPG liposomes, fully blocked the oxidation of CL (Figures 9 and 10(a)). Apparently, amphiphilic antioxidants could easily escape the POPG liposomes to interact with the CL liposomes or the MeO-AMVN radicals in the solution.

As noted in Introduction, SkQ ions and other amphiphilic antioxidants exhibited pronounced therapeutic effects* in vivo* [20, 44, 52, 54, 55, 61–74, 127–129], in spite of high levels of hydrophilic and hydrophobic antioxidants present in the cells, even when added in minute amounts. The mechanism of this specific therapeutic action has remained obscure. Here we show that natural ubiquinols extremely efficiently protect CL from oxidation within membrane bilayer, so that their failure* in vivo* cannot be attributed to poor antioxidant qualities. The reported particular efficiency of amphiphilic antioxidants [20, 44, 52, 54, 55, 61–74, 127–129] might be related to our observation that amphiphilic antioxidants, unlike the hydrophobic ones, can protect CL molecules from the water phase (Figure 9). Indeed, given that many CL molecules are trapped within respiratory enzymes [22], as well as between respiratory enzymes of supercomplexes [26–28], that is, close to locations where ROS are generated [9, 33–40, 45], it is tempting to anticipate that these trapped CL molecules might be the first to become accessible to ROS. This fraction of CL molecules, however, might be secluded both from the bulky ubiquinol-10 molecules with their large isoprenoid tails confined between the membrane leaflets [130] and from the highly polar water-soluble antioxidants, such as glutathion. Earlier we have suggested that these secluded CL molecules could be still accessible to small, nimble, and amphiphilic artificial antioxidants [19, 122]. The data in Figure 9 indicate that a further advantage of amphiphilic antioxidants over natural ubiquinols and tocopherols is the ability to reach their “occluded” CL targets from the water phase, without the need to get through tightly packed *α*-helices of respiratory supercomplexes. Such a scenario would explain the ability of amphiphilic antioxidants to prevent the CL oxidation* in vivo*.

Hence, those CL molecules that are occluded within respiratory complexes are close to the sources of ROS and should be the first targets of oxidation in response to a redox imbalance in the respiratory chain. The same secluded CL molecules should be hardly accessible to natural antioxidants. Taken together, these two factors may explain why CL molecules are those preferably oxidized upon oxidative stress [20, 43, 44]. An interaction with cytochrome *c* molecules at the membrane surface would result in a further oxidation of CL and release of cytochrome *c* into the cytoplasm [41–44]. Hence, the very susceptibility of CL molecules to oxidation should accelerate the delivery of the message on a redox disaster within mitochondria to the cytoplasmic apoptotic machinery, which might explain the recruiting of CL as the primary sensor of the apoptotic cascade by nature.

In sum, amphiphilic, positively charged antioxidants targeted to mitochondria might specifically protect the occluded CL “sensors” from oxidation and postpone thereby apoptosis. Such postponement might be life-saving in case of acute but transient oxidative stress, such as upon traumas or strokes. Therefore amphiphilic antioxidants, even being added in minute amounts over a huge background of natural ubiquinol and tocopherol, might be efficient in preventing irreversible damage and death.

## 5. Conclusions

We found that natural ubiquinols were as efficient in protecting CL liposomes from peroxidation as ubiquinol-based amphiphilic antioxidants, whereas chromanol-based antioxidants were 2-3 times less efficient. The amphiphilic antioxidants, but not the natural ubiquinols and *α*-tocopherol, could, additionally, interact with the CL bilayer from the water phase. Building on these data, we suggest that the therapeutic efficiency of mitochondrially targeted amphiphilic antioxidants is owing to their ability to protect those CL molecules that are inaccessible to natural hydrophobic antioxidants, being trapped within respiratory supercomplexes. The high susceptibility of such occluded CL molecules may have prompted their recruitment as the sensors of oxidative stress by nature.

## Figures and Tables

**Table 1:** Relative efficiencies of various antioxidants in CL liposomes (note the split *y*-axis). The values for oxidation rates were obtained from the data in Figures 6, 7 [Fig fig8] [Fig fig9], and 10 and analogous repetitive experiments as described in Section 2. The chain propagation length for pure CL and each antioxidant was calculated using the radical production rate of 11.8 nM/min, as derived from the *α*-tocopherol run; see Section 4. The inhibition efficiencies were calculated as ratios of the respective uninhibited and inhibited oxidation rates. The inhibition duration rates were determined from the data in Figures 5 [Fig fig6]–7 and 10.

Antioxidant	CL oxidation rate, nM/min	Chain propagation length	Inhibition efficiency	Inhibition duration (min)
Pure CL	334 ± 32	28	—	—
decPQH_2_	3.28 ± 1.1	0.28	102	48.7 ± 2.1
SkQ1H_2_	3.87 ± 0.6	0.33	86	58.7 ± 5.7
decUQH_2_	4.49 ± 1.2	0.38	74	114 ± 4.4
MitoQH_2_	6.70 ± 2.0	0.57	50	91 ± 8.9
Q_6_H_2_	4.05 ± 0.5	0.34	82	124 ± 9
Q_10_H_2_	4.55 ± 0.4	0.39	73	104 ± 1
*α*-Tocopherol	10.6 ± 0.5	0.90	32	171 ± 1
HPMC	8.62 ± 1.8	0.73	39	N.A.^*∗*^

^*∗*^The duration of action varied dramatically in the case of HPMC because of its variable loss upon the incorporation into liposomes; see Section 4.

## Abbreviations

CL: Cardiolipin
decPQH_2_: Decylplastoquinol
decUQH_2_: Decylubiquinol
HPMC: 6-Hydroxy-2,2,5,7,8-pentamethylbenzochroman
MeO-AMVN: 2,2′-Azobis(4-methoxy-2,4-dimethylvaleronitrile)
MitoQH_2_: 10-(6′-Ubiquinonyl)decyltriphenylphosphonium
MPT: Mitochondrial permeability transition
LDL: Low density lipoprotein
POPG: 1-Palmitoyl-2-oleoyl-phosphatidylglycerol
Q_6_H_2_: Ubiquinol-6
Q_10_H_2_: Ubiquinol-10
ROS: Reactive oxygen species
SkQ: Compounds composed of penetrating cation and quinone
SkQH_2_: Reduced (quinol or hydroquinone) forms of SkQ
SkQ1H_2_: 10-(6′-Plastoquinonyl)decyltriphenylphosphonium
SQ or QH^*∙*^: Semiquinone.
